# Thinness and fecundability: Time to pregnancy after adolescent marriage in rural Bangladesh

**DOI:** 10.1111/mcn.12985

**Published:** 2020-03-24

**Authors:** Jinhee Hur, Keith P. West, Abu Ahmed Shamim, Mahbubur Rashid, Alain B. Labrique, Lee S.F. Wu, Hasmot Ali, Barkat Ullah, Kerry J. Schulze, Rolf D.W. Klemm, Parul Christian

**Affiliations:** ^1^ Center for Human Nutrition, Department of International Health Johns Hopkins Bloomberg School of Public Health Baltimore Maryland; ^2^ The JiVitA Project Gaibandha Bangladesh; ^3^ Department of Nutrition University of California, Davis Davis California

**Keywords:** adolescent girls, arm circumference, Bangladesh, fecundability, pregnancy, South Asia, undernutrition

## Abstract

Undernutrition may affect fecundability, but few studies have quantified this relationship. In rural Bangladesh, where newlywed couples face strong pressures to become pregnant, we assessed fecundability, estimated by time to pregnancy (TTP), and its association with preconceptional thinness among nulligravid, newlywed female adolescents. During 2001–2002, 5,516 newlywed women aged 12–19 years participated in a home‐based, 5‐weekly surveillance system for 5–6 years to enrol pregnant women into an antenatal vitamin A or β‐carotene supplementation trial. Thinness was defined as a left mid‐upper arm circumference (MUAC) ≤21.5 versus >21.5 cm. At each visit, staff obtained a monthly history of menstruation. Report of amenorrhea prompted a human chorionic gonadotropin urine test to confirm pregnancy. We derived hazard ratios (with 95% confidence intervals [CI]) for pregnancy and Kaplan–Meier curves for TTP. Ages of women at marriage and pregnancy detection (mean ± standard deviation) were 15.3 ± 1.9 and 17.0 ± 1.9 years, respectively. A total of 82.7% of thinner and 87.3% of better nourished women became pregnant. The unadjusted and multivariable relative hazard of ever becoming pregnant was 0.84 (95% CI [0.78, 0.89]) and 0.86 (95% CI [0.81, 0.92]), respectively, and TTP was 12 weeks longer (median [95% CI]: 63 [58–68] vs. 51 [49–54]) in women whose MUAC was ≤21.5 versus >21.5 cm. In rural Bangladesh, thin adolescent newlywed girls have a lower probability of becoming pregnant and experience a longer time to pregnancy.

**Key messages**

- In rural Bangladesh, where newlywed couples face strong social pressures to become pregnant, undernourished newlywed adolescent girls with a preconceptional mid‐upper arm circumference ≤21.5 cm are less likely to become pregnant in the first ~5 years of marriage.
- Among those who become pregnant, thinner adolescent girls are more likely to experience delayed conception.
- Explanations could be biological, but social reasons may also exist.

**List of Abbreviations**
BMI: body mass index
CI: confidence interval
GnRH: gonadotropin releasing hormone
hCG: human chorionic gonadotropin
HR: hazard ratio
MUAC: mid‐upper arm circumference
SD: standard deviation
SES: socioeconomic status
TTP: time to pregnancy

## 1 INTRODUCTION

In rural Bangladesh, most women marry as adolescents (National Institute of Population Research and Training & ICF, 2019) and face strong societal pressures to become pregnant, leading to high pregnancy rates in the first few years of marriage (Sayem & Nury, 2011). Adolescent pregnancy may both limit a young woman's remaining growth potential (Rah et al., 2008) and increase the risk of adverse birth outcomes (Kozuki et al., 2013). One explanation for increased health risks of adolescent pregnancy may be poor preconceptional nutritional status of a young woman, a particular concern in rural Bangladesh where adolescents tend to be stunted and thin (Rah et al., 2008), emphasizing the importance of taking policy steps toward reducing early teen marriage and pregnancy in South Asia, as recommended by the United Nations Children's Fund (2014) and recently enacted in Bangladesh (Government of the People's Republic of Bangladesh Ministry of Women and Child Affairs, 2017). Although an important step forward, a limitation of the legislation lies in a waiver to marry under 18 years of age when parental consent exists.

Preconceptional undernutrition may also affect fecundability of women, an issue that has received little recent attention. Forty‐six years ago, the association between nutritional status, body composition, and reproductive performance of women was extensively explored by Frisch and McArthur (1974), who postulated that the human body requires a critical mass and proportion of body fat to achieve menarche and maintain regularity of ovulatory and menstrual cycles. Risk of reproductive failure has also been observed to rise during famine (Stein & Susser, 1975). A small body of literature exists across diverse cultural settings on the association between maternal body mass index (BMI) and fecundability, defined as the probability of conception under conditions of regular sexual activity during a normal menstrual cycle in the absence of contraception (United Nations, 1958). In general, these studies in diverse settings suggest that women with either an extremely low (<18.5) or high (≥30) BMI have low fecundability (Bolumar, Olsen, Rebagliato, Saez‐Lloret, & Bisanti, 2000; Gesink Law, Maclehose, & Longnecker, 2007; Hassan & Killick, 2004).

Mid‐upper arm circumference (MUAC) is a summary measure of cross‐sectional arm adiposity, musculature, fascia, and humoral bone (Gibson, 2005). As such, it is considered a simple and quantitative anthropometric indicator of current nutritional status that is correlated with BMI (Gibson, 2005) and detects a spectrum of status, ranging from wasting (or thinness) (James et al., 1994) to overweight and obesity (Craig, Bland, Ndirangu, & Reilly, 2014). In impoverished populations, it is of interest that a decreasing MUAC during pregnancy has been shown to be associated, in a dose‐response manner, with risk of maternal mortality (Christian et al., 2008). Moreover, maternal thinness has been known to increase risks of adverse pregnancy outcomes, such as a preterm birth (Feresu, Harlow, & Woelk, 2004), a small‐for‐gestational‐age birth and low birth weight (Sebayang et al., 2012), that may also perpetuate an intergenerational cycle of undernutrition in undernourished populations (United Nations et al., 1992). Although MUAC is often used to assess maternal nutritional status and examined in relation to pregnancy outcomes, to our knowledge, the hypothesis that a low preconceptional MUAC increases risk of infecundability remains to be examined in modern, nonfamished adolescent populations such as those typically existing in rural South Asia.

Within the context of an earlier population‐based, routine pregnancy surveillance system in rural northwest Bangladesh, we have defined the indicator time to pregnancy (TTP) as a proxy for fecundability to prospectively assess the probability of conception by nutritional status in adolescent nulligravid newlyweds, among whom contraception is rarely practiced due to high social expectations to become pregnant. We present population‐based evidence that is consistent with a hypothesis that thinness in nulligravid adolescent women lowers fecundability.

## 2 METHODS

### 2.1 Study population

This prospective study was conducted in 19 rural unions (lowest administrative unit of local government) of Gaibandha and Southern Rangpur Districts, an area of ~435 km^2^ that is typical of northwestern Bangladesh with a population of ~650,000 people (Labrique et al., 2011). Participants for this study comprised adolescent girls <20 years of age who were married for 4 or fewer months at the time they were enlisted into a home‐based pregnancy surveillance system designed to detect and recruit women in the first trimester of pregnancy into a double‐masked, cluster‐randomized, placebo‐controlled trial of weekly vitamin A or β‐carotene supplementation (JiVitA‐1 Trial; West et al., 2011). The trial was conducted from August 2001 to January 2007 and registered on ClinicalTrials.gov (NCT00198822). Only data for newlyweds identified and placed under 5‐weekly, home‐based pregnancy surveillance in 2001 and 2002 (*n* = 6,281 women) were eligible for this analysis to maximize the duration over which enlisted women could become pregnant (i.e., 5–6 years) and minimize effects of early censoring on estimates of pregnancy incidence and time to pregnancy. Verbal‐informed consent was obtained from newlywed women at enrolment into the pregnancy surveillance system and before pregnancy testing, documented by field staff initializing data collection forms. Given their marital status, women under 18 years of age were considered emancipated minors and free to consent or refuse participation without parental concurrence.

### 2.2 Data collection

Starting at the outset of the trial in 2001, over 110,000 digitally mapped and addressed homes were initially visited to enrol married, non‐pregnant women of reproductive age into the pregnancy surveillance system, recording age, type of family planning method currently practiced, and left MUAC to the nearest 0.1 cm using a Zerfas insertion tape (Zerfas, 1975). Circumferential measurement was recorded in triplicate, accepting the median as true value. Thereafter, homes were visited every 5 weeks by 596 local resident staff to obtain a menstrual history over the previous month and administer a urinary human chorionic gonadotropin (hCG) test to women reporting amenorrhea to confirm pregnancy. Test‐positive, consenting women were enrolled into the antenatal supplementation trial. During each 5‐week round, all households in the study area were also canvassed to enlist any newly married women (married ≤4 months) for pregnancy surveillance, for whom age, date of marriage, family planning method, and MUAC were also recorded. The system ensured that newly married young women were continuously entering into the pool of eligible trial participants and followed for menstrual history every 5 weeks. Shortly after pregnancy ascertainment, usually in the first trimester, a risk factor interview was conducted by trained staff that included a 7‐ and 30‐day history of morbidity symptoms, 7‐day food frequency and recall of work performed and household demographic characteristics, and socioeconomic factors that allowed derivation of a living standards index (Gunnsteinsson et al., 2010) among women who contributed a pregnancy to the trial.

### 2.3 Study terms and definitions

Adolescence was defined to comprise age 12 to 19 years, inclusively. A MUAC ≤21.5 cm obtained at time of enlistment defined thinness, corresponding to the ~25th percentile value of the distribution of MUAC across all women enrolled into the JiVitA‐1 trial and a cutoff previously reported to describe wasting undernutrition (Kim et al., 2012). A higher MUAC (>21.5 cm) was considered to reflect normal status. Pregnancy was defined by a history of amenorrhea in the previous month followed by a confirmatory, positive hCG urine test (Clue; Orchid, Pune, India). We defined TTP as the duration between the calendar weeks of marriage and reported last menstrual period. Assuming couples were sexually active, fecundability, formally defined as the probability of conceiving within a single menstrual cycle (United Nations, 1958), was approximated by TTP, as has been used elsewhere (Jensen, Scheike, Keiding, Schaumburg, & Grandjean, 1999). As a corollary, an undetected pregnancy among adolescent newlyweds over the 5‐ to 6‐year period of routine pregnancy surveillance has been considered an indication of infecundability.

### 2.4 Exclusion criteria

**Figure 1:**
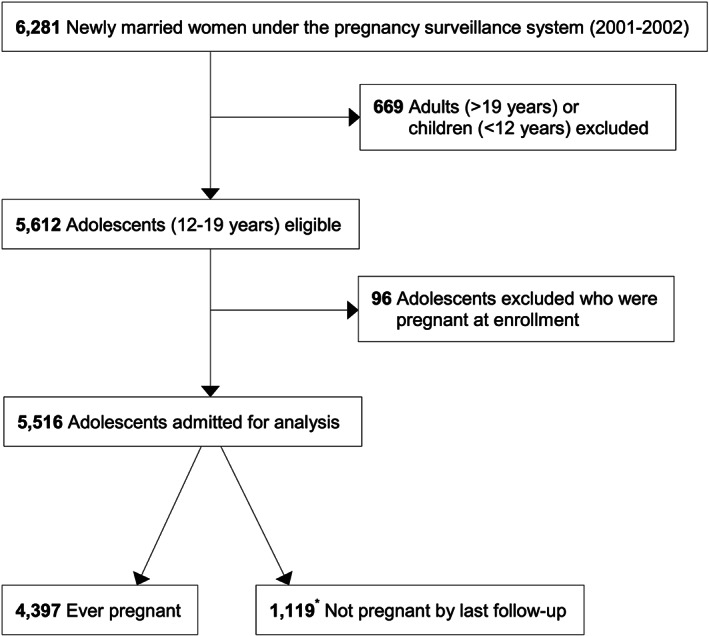
Flow diagram of the study participants. ^*^ *n* = 248 followed through study censure (260‐316 weeks; ≥5 years), *n* = 471 followed for ≥104–259 weeks (2–4.99 years) and *n* = 400 followed for <104 weeks (<2 years)

Of 6,281 newly married women enrolled for pregnancy surveillance during 2001–2002, 669 were excluded for being outside our defined adolescent age range, 96 of whom were recorded as <12 years of age and thus of uncertain fertility and 573 being >19 years (Figure 1). Adolescent newlyweds ascertained to be already pregnant at enlistment for surveillance (*n* = 96) were also excluded, leaving data for 5,516 newly married, nulligravid adolescent women to be analysed for this study.

### 2.5 Statistical analysis

Descriptive data were presented as means with standard deviations (SD) for normally distributed continuous variables and frequencies (%) for categorical variables. Differences in baseline characteristics between the two MUAC groups were analysed by *t* test for continuous variables and chi‐squared test for categorical variables. The number of new pregnancies and person‐time (in weeks) was examined among women with a lower vs higher preconceptional MUAC. We used simple and multivariable Cox proportional hazards models, the latter adjusting for marital age, calendar year and season of marriage, and reported contraceptive use at enlistment, to evaluate the relative hazard (with 95% confidence interval [CI]) of becoming pregnant by duration since marriage among thin vs better nourished women. Although not significantly different between the MUAC groups, calendar year and season of marriage were kept in the multivariable models as variables of generic interest for adjustment. We present Kaplan–Meier curves for TTP by preconceptional MUAC, overall and by age groups of 12–13, 14–15, and 16–19 years, representing early, mid‐, and later postmenarcheal adolescence, respectively. From the curves, the median survival time with 95% CI (expressed in weeks) was calculated as the time by which 50% of the population had become pregnant. We also present a Kaplan–Meier curve for TTP by preconceptional MUAC, restricting data to newlyweds who ever became pregnant during a 5‐ to 6‐year surveillance period (*n* = 4,397). As supplementary analyses, we examined the dose‐response association between thinness and fecundability at progressively lower preconceptional MUAC cutoffs of ≤20 versus >20 and ≤19 versus >19 cm, as permitted by strata sample sizes. For the entire analytic population and those who never became pregnant during the period of observation, the calendar year and month of the entry into the pregnancy surveillance system and time to censorship were compared between the two MUAC groups, respectively. As sensitivity analyses, the multivariable Cox proportional hazards model was fit only among those who reported practicing no family planning at enlistment. As the primary analysis included all women confirmed pregnant by urine testing, we also repeated the TTP analysis excluding women who subsequently miscarried. Correlation between MUACs measured at shortly after marriage and first trimester was checked. Using a linear regression model of TTP on preconceptional MUAC, we took consideration of household socioeconomic status (SES) into analysis, as assessed by a living standards index (Gunnsteinsson et al., 2010) derived from questions asked in the first trimester of pregnancy. All tests were two‐tailed with a significance level of *α* = .05. All statistical analyses were conducted using STATA 14 (StataCorp LP, College Station, Texas, USA).

### 2.6 Ethical considerations

Procedures of data collection and consent were approved by the Institutional Review Board at the Johns Hopkins Bloomberg School of Public Health, Baltimore, MD, and the Bangladesh Medical Research Council, Dhaka, Bangladesh.

## 3 RESULTS

### 3.1 Characteristics of study participants

Among 5,516 newly married adolescent girls aged 12 to 19 years, the ages at marriage and first pregnancy detection (mean ± SD) were 15.3 ± 1.9 and 17.0 ± 1.9 years, respectively (Table 1). For 3,746 and 1,770 adolescent wives whose initial MUAC was >21.5 and ≤21.5 cm, the corresponding ages at marriage and pregnancy, indicated by reported last menstrual period, were 15.5 ± 1.8 and 17.2 ± 1.9 years and 14.9 ± 1.9 and 16.7 ± 1.9 years (*p* < .001 for both comparisons), respectively. Preconceptional MUAC was 22.6 ± 1.9 cm in women who became pregnant and 22.2 ± 2.0 cm in those who remained under observation and free of pregnancy for the entire 5‐ to 6‐year study period (*p* < .01). Most (89.7%) women reported practicing no family planning methods at enlistment for surveillance. Among those who did, oral contraceptive use was the most common method (8.9%). Year and season of marriage were comparable between the MUAC groups.

**Table 1:** Characteristics of Bangladeshi adolescent newlyweds by mid‐upper arm circumference (MUAC) at enrolment into the pregnancy surveillance system during the JiVitA‐1 trial, 2001–2002 enlisted cohort

Variable	All (*n* = 5,516)		MUAC >21.5 cm (*n =* 3,746)		MUAC ≤21.5 cm (*n =* 1,770)		*p* a
	*n*	%	*n*	%	*n*	%	
Age at marriage (years)^b^							<.001
12	311	5.6	120	3.2	191	10.8	
13	736	13.3	447	11.9	289	16.3	
14	1,058	19.2	694	18.5	364	20.6	
15	1,076	19.5	765	20.4	311	17.6	
16	789	14.3	569	15.2	220	12.4	
17	761	13.8	569	15.2	192	10.9	
18	492	8.9	357	9.5	135	7.6	
19	293	5.3	225	6.0	68	3.8	
Mean	15.3		15.5		14.9		
*SD*	1.9		1.8		1.9		
Age at pregnancy (years)							<.001
*n*	4,397		3,056		1,341		
Mean	17.0		17.2		16.7		
*SD*	1.9		1.9		1.9		
MUAC at enlistment (cm)							<.001
Mean	22.5		23.5		20.4		
*SD*	1.9		1.4		0.9		
Family planning practice at enlistment^b^^,^ c							<.001
No family planning	4,947	89.7	3,291	87.9	1,656	93.7	
Oral pills	489	8.9	392	10.5	97	5.5	
Condoms	72	1.3	58	1.6	14	0.8	
Natural method	3	0.1	2	0.1	1	0.1	
Intrauterine device	1	0.0	1	0.0	0	0.0	
Injectable contraception	1	0.0	1	0.0	0	0.0	
Other	1	0.0	1	0.0	0	0.0	
Year of marriage							.321
2001	1,637	29.7	1,096	29.3	541	30.6	
2002	3,879	70.3	2,650	70.7	1,229	69.4	
Season of marriage^b^							.676
Monsoon	2,363	42.8	1,624	43.4	739	41.8	
Post‐monsoon	1,048	19.0	707	18.9	341	19.3	
Post‐harvest	1,139	20.7	771	20.6	368	20.8	
Hot and humid summer	966	17.5	644	17.2	322	18.2	
Living standards index^d^							<.001
<Median	1,648	37.5	1,080	35.3	568	42.4	

Abbreviation: MUAC, mid‐upper arm circumference (cm).

a Differences between the two MUAC groups were analysed by *t* test for continuous variables and chi‐squared test for categorical variables.

b Percentages may not equal to 100% due to rounding.

c Missing values (*n* = 2 for MUAC ≤21.5 cm) were excluded from the denominator calculating frequency distributions.

d A composite index describing the socioeconomic status of a household with −0.259 representing the median value in the placebo group of the JiVitA‐1 trial (West et al., 2011), which was measured only among women who became pregnant; missing values (*n* = 690 and *n* = 429 for MUAC >21.5 versus ≤21.5 cm, respectively) were excluded from the denominator calculating frequency distributions.

### 3.2 MUAC and time to pregnancy

**Figure 2:**
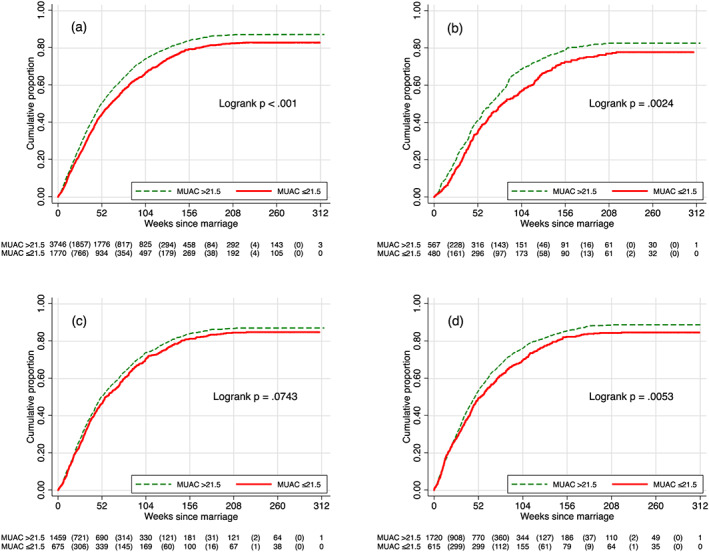
Cumulative proportion of women becoming pregnant since marriage stratified by age group and mid‐upper arm circumference (MUAC ≤21.5 vs. >21.5 cm) at the time of enrolment into the pregnancy surveillance system shortly after marriage. (a) All ages (*N* = 5,516); (b) aged 12–13 years (*n* = 1,047); (c) 14–15 years (*n* = 2,134); and (d) 16–19 years (*n* = 2,335). The table under each plot presents the number of adolescent, non‐pregnant newlyweds at each time point who were susceptible to pregnancy the following year and (in parentheses) the number of newly detected pregnancies during each subsequent 52‐week (1‐year) interval

Adolescent newlyweds were under follow‐up for the average period of 76.2 ± 73.1 weeks, and the longest follow‐up period was 317 weeks (i.e., 6.1 years). The overall TTP from marriage was 53.4 ± 43.1 weeks. Cumulatively, 48.4%, 71.6%, and 82.3% of women became pregnant within 52, 104, and 156 weeks of follow‐up, respectively (Figure 2a). The hazard rate of first pregnancy was significantly lower, by 16%, among adolescents with a preconceptional MUAC ≤21.5 versus >21.5 cm (hazard ratio [HR] 0.84, 95% CI [0.78, 0.89], *p* < .001; Table 2). Significant HRs with 95% CIs excluding 1 were observed for women who were 12, 16, and 17 years of age at marriage. The difference in the likelihood of becoming pregnant remained in a multivariable Cox proportional hazards model, in which newlyweds with an initial MUAC ≤21.5 cm were 14% less likely to ever become pregnant than those with a larger MUAC, after adjusting for marital age, calendar year and season of marriage, and initial contraceptive practice at enlistment, (HR 0.86, 95% CI [0.81, 0.92], *p* < .001; Table 3). In a dose‐response analysis, the HR of ever becoming pregnant decreased to 0.79 (95% CI [0.70, 0.88], *p* < .001) and 0.78 ([0.65, 0.95], *p* = .012) for women with an early postnuptial MUAC of ≤20 versus >20 and ≤19 versus >19 cm, respectively (Tables S1 and S2, respectively).

**Table 2:** Unadjusted hazard ratio (HR) of pregnancy in Bangladeshi adolescent newlyweds by age at marriage and early postnuptial mid‐upper arm circumference (MUAC) during 5–6 years of observation of the JiVitA‐1 trial, 2001–2002 enlisted cohort

Age at marriage (years)	Total *n*	MUAC >21.5 cm			MUAC ≤21.5 cm			HR	95% CI	*p*
		Total	Pregnant	Person‐weeks^a^	Total	Pregnant	Person‐weeks^a^			
Overall	5,516	3,746	3,056	272,919	1,770	1,341	147,428	0.84	[0.78, 0.89]	<.001
12	311	120	90	10,807	191	122	19,449	0.74	[0.57, 0.98]	.034
13	736	447	343	37,360	289	209	26,648	0.86	[0.73, 1.03]	.094
14	1,058	694	558	54,675	364	283	29,609	0.93	[0.80, 1.07]	.297
15	1,076	765	631	53,841	311	245	24,038	0.91	[0.78, 1.05]	.191
16	789	569	472	39,327	220	173	18,243	0.84	[0.70, 1.00]	.047
17	761	569	479	36,016	192	149	14,691	0.81	[0.67, 0.97]	.024
18	492	357	300	24,959	135	105	10,520	0.87	[0.70, 1.09]	.231
19	293	225	183	15,934	68	55	4,229	1.10	[0.81, 1.49]	.543

a Person‐weeks: total person‐time of observation, overall, and by age at marriage.

**Table 3:** Adjusted hazard ratio (HR) of pregnancy in Bangladeshi adolescent newlyweds by early postnuptial mid‐upper arm circumference (MUAC) during 5–6 years of observation of the JiVitA‐1 trial, 2001–2002 enlisted cohort

Variable	Pregnancy (*n*)	HR	95% CI	*p*
MUAC at enlistment (cm)				<.001
>21.5 (*n* = 3,746)	3,056	1.00		
≤21.5 (*n* = 1,768)^a^	1,340	0.86	[0.81, 0.92]	
Family planning practice at enlistment				.562
No	3,929	1.00		
Yes	467	0.97	[0.88, 1.07]	
Age at marriage (years)	4,396	1.06	[1.04, 1.07]	<.001
Year of marriage				.024
2001	1,354	1.00		
2002	3,042	0.92	[0.86, 0.99]	
Season of marriage				.084
Monsoon	1,919	1.00		
Post‐monsoon	829	0.99	[0.91, 1.07]	
Post‐harvest	914	1.07	[0.99, 1.16]	
Hot and humid summer	734	0.95	[0.87, 1.04]	

a Missing values: *n* = 2, due to missing data in family planning practice covariate.

Adolescents with a MUAC ≤21.5 cm took, on average, 11.6 weeks longer to become pregnant than those with a MUAC >21.5 cm (median [95% CI]: 62.9 [58.0–68.1] vs. 51.3 [49.0–54.3] weeks, respectively, *p* < .001; Figure 2a). The difference was statistically significant within age‐at‐marriage strata of 12–13 and 16–19 years (Figure 2b,d) and especially prominent in the youngest ages. With older age at marriage, the median time to pregnancy was reduced, and the cumulative proportion of women who became pregnant increased more rapidly, irrespective of MUAC. Within each depicted age group, the proportion of women meeting our definition of infecundability, as having not become pregnant during their observed periods of follow‐up, was higher in less well‐nourished than better nourished women: 22.2%, 15.3%, and 15.5% versus 17.3%, 12.9%, and 11.1%, respectively.

**Figure 3:**
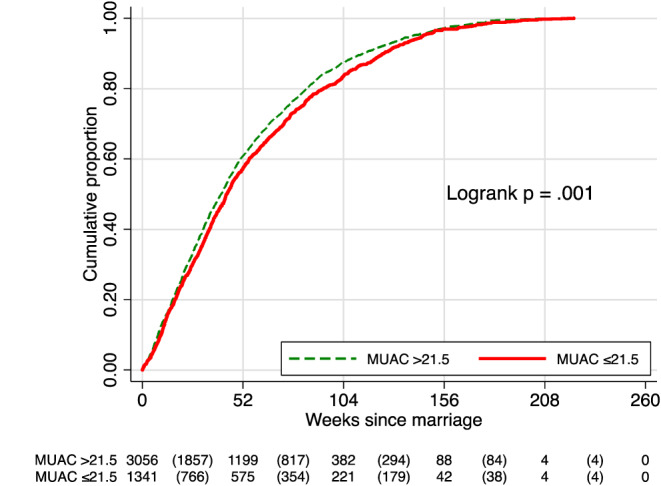
Cumulative proportion of women becoming pregnant since marriage stratified by mid‐upper arm circumference (MUAC ≤21.5 vs. >21.5 cm) at the time of enrolment into the pregnancy surveillance system shortly after marriage, only among those who ever became pregnant during a 5‐ to 6‐year surveillance period (*n* = 4,397). The table under the plot presents the number of adolescent, non‐pregnant newlyweds at each time point who were susceptible to pregnancy the following year and (in parentheses) the number of newly detected pregnancies during each subsequent 52‐week (1‐year) interval

Restricting analysis to only those who became pregnant, an additional Kaplan–Meier curve for TTP showed a trend of a longer, but less obvious, TTP among adolescent newlyweds with a thinner than larger preconceptional MUAC (*p* = .001; Figure 3). This signalled most of the difference in TTP by MUAC could be attributed to those never becoming pregnant over the 5‐ to 6‐year period of surveillance, given comparable distributions of entry into the study and time to censorship between the two MUAC groups (Tables S3 and S4).

### 3.3 Sensitivity analyses

Excluding either the ~10% of women from the study population who reported any contraceptive use at enlistment (*n* = 567) or the ~8% of women who reportedly miscarried (*n* = 453), led to virtually the same HR findings (data not shown). Also, MUAC measured at enlistment and after pregnancy detection (usually first trimester) was strongly correlated (*r* = .67, *p* < .001; data not shown) with the odds ratio of a MUAC remaining ≤21.5 cm in early pregnancy, given a MUAC at enlistment of ≤21.5, and was 8.99 (95% CI [7.68, 10.53], *p* < .001; data not shown), reflecting persistence in nutritional status over the period of time extending from shortly after marriage through early pregnancy. Of newlyweds who ever became pregnant (*n* = 4,397), household SES, reflected by a composite living standards index (Gunnsteinsson et al., 2010), was not associated with TTP and did not change the association between preconceptional MUAC and TTP when introduced as a covariate into a linear regression model (data not shown).

## 4 DISCUSSION

This study found that thinness is associated with a delay in time to pregnancy in a largely noncontracepting population cohort of nulligravid, newlyweds, 12 to 19 years of age, who entered marriage in 2001–2002 in rural Bangladesh. Specifically, a left MUAC ≤21.5 cm, indicating thinness and measured within 4 months of marriage, was associated with a 16% lower probability of becoming pregnant over the first 5 to 6 years of marriage than young women from the same area whose arm circumference was larger shortly after marriage. Adjusting for several associated factors, the HR remained stable at 0.86, reflecting a 14% lower probability of becoming pregnant. The association was observed across all age strata, significantly so for adolescent wives who were 12–13 and 16–19 years of age at marriage, and appeared to be dose‐responsive in that women with a postnuptial arm circumference of 21.5, 20, and 19 cm or less were 14%, 21%, and 22% less likely, respectively, to become pregnant than better nourished women (above these cut‐points). After the first 3 years of marriage and surveillance, the cumulative probability of becoming pregnant for thin and normally nourished wives plateaued over the next 3–4 years at 82% and 87%, respectively. Second, among participants who did become pregnant over the observed time period, thinner newlyweds were marginally but significantly slower to become pregnant than their better nourished peers. These findings are novel with respect to the size of the population cohort under surveillance; the length of time followed; use of standardized methods deployed to regularly monitor pregnancy, which included hCG urine testing to confirm suspected pregnancies initially detected by a 5‐weekly history of amenorrhea; and MUAC deployed as an indicator of nutritional status typically measured during the first 4 months of marriage.

Our findings are consistent with earlier reports in human populations revealing that undernutrition, measured as a low BMI, may affect fecundity, referring to reproductive capability. In studies conducted in Europe and the United States, women with a BMI <19 kg/m^2^ exhibited delayed fecundability (Bolumar et al., 2000; Gesink Law et al., 2007; Hassan & Killick, 2004; Zaadstra et al., 1993), defined as achieved conception under conditions of frequent intercourse within the time period of a menstrual cycle (United Nations, 1958), thus implicating a suppressive role of chronic undernourishment on conception. The present study did not assess within‐menstrual cycle conception but rather employed a summed expression of fecundability over a period of 5–6 years in a minimally contracepting, nulligravid population of newlywed couples facing strong societal pressures to bear children soon after marriage (Sayem & Nury, 2011).

The observed findings may reflect effects of undernutrition on reproductive biology or influences of social and economic status on nutritional status, age at marriage, and tempo of conception among newly married couples in rural Bangladesh. Biologically, delayed reproductive readiness could be consequent to physiological stresses imposed by chronic undernutrition, common among rural South Asian children, adolescents, and adult women (NCD Risk Factor Collaboration, 2017), acting to defer or diminish reproductive competence that ensues with puberty (Gluckman & Hanson, 2006). Fecundity is considered to be achieved through the pubescent period, commensurate with menarche, via a cascade of orchestrated hormonal signals, involving release of hypothalamic gonadotropin releasing hormone (GnRH), gonadotropins and sex steroid hormones that are triggered and dependent on adequate energy nutriture of a young woman (Abreu & Kaiser, 2016). Given that a specific level of GnRH expression is required to maintain normal pulse of luteinizing hormone, reduced GnRH may result in irregular menstruation or amenorrhea and may adversely affect ovulation and reproductive health (Filicori et al., 1993). Undernutrition can induce the reduced levels of GnRH (Warren, 1983), and women who have lost excessive weight have been found to exhibit reduced secretion and responsiveness of luteinizing hormone to GnRH, a condition seen in prepubertal girls (Warren et al., 1975).

Altered endocrine function resulting from malnutrition, either a deficit or excess in energy intake, may be communicated to the reproductive axis in the higher brain centres via endocrine factors that are regulated by body composition. Leptin, produced by adipose tissues and a signal of positive energy balance (Tena‐Sempere, 2007), and insulin and insulin‐like growth factor‐1, both sensitive to nutritional status (Donahue & Phillips, 1989), are known to have excitatory actions on the GnRH neurons (Barr, Malide, Zarnowski, Taylor, & Cushman, 1997; Wolfe, Divall, & Wu, 2014). Clinical studies of treatment with such hormones support their critical role in promoting normal reproductive functions, including secretion of gonadotropins, ovulation, menstrual regulation, and pubertal maturation in humans (von Schnurbein et al., 2012) and animals (Hiney, Srivastava, & Dees, 2004; Hiney, Srivastava, Nyberg, Ojeda, & Dees, 1996). These insights support a putative role for undernutrition in disrupting fecundity through dysregulation of neuroendocrine function that could plausibly render undernourished young women less able to conceive or carry a pregnancy.

However, social factors may also have influenced our observed differences in time to pregnancy by bridal nutritional status. In some studies conducted in Bangladesh, age at marriage has been observed to be inversely related to weight among adolescent girls, attributed to greater attraction and desirability of a well‐nourished and healthier girl (Riley, 1994), which could also be a factor that shortens time to conception. On the other hand, in rural Bangla society, thinner women are more likely to live in poorer households (Gunnsteinsson et al., 2010), which may be more inclined to enter their daughter into marriage at an earlier age to both improve her economic security and secure a more favourable financial arrangement for a bride's family (Bates, Schuler, Islam, & Islam, 2004). A study analysing data from the Bangladesh Demographic and Health Surveys during the previous decade indicates that adolescent girls with poorer SES and lower educational attainment are more likely to experience adolescent motherhood, possibly a consequence of early marriage and social pressure of childbearing in the first postnuptial year (Islam, Islam, Hasan, & Hossain, 2017). Given that we did not collect information on such social factors that may be associated with adolescent pregnancy represents a limitation of our study, as such socioeconomic concerns may have motivated an earlier age of marriage among less well‐nourished young women living in poorer households compared with peers of both better nutritional and socioeconomic status in our study population. Notwithstanding the consequent earlier age of conception among thinner than heavier nulligravid newlyweds, amidst presumably comparable societal pressures to have children, time to pregnancy was longer, including a lower likelihood of becoming pregnant at all during the first 5–6 years of marriage, in thinner than heavier women.

Only among those who become pregnant could we consider household SES in the time‐to‐pregnancy analysis, as assessed by a living standards index (Gunnsteinsson et al., 2010) derived from questions asked in the first trimester of pregnancy. Household SES was neither associated with time to pregnancy independently nor changed the association between preconceptional MUAC and time to pregnancy, preserving the longer time to conceive among undernourished brides. Although socioeconomic data were not available among women who never contributed a pregnancy to the study, this finding indirectly suggests that differences in SES may not have influenced the higher risk of never becoming pregnant among thinner wives, observed during the follow‐up.

Other potential reasons for underlying infecundability, a phenomenon applicable to both females and males, include a blocked fallopian tube or low sperm count (Thurston, Abbara, & Dhillo, 2019), although these could be anticipated to occur regardless of girls' MUAC status. Undernutrition can also be a by‐product of poverty (United Nations Children's Fund, 1990), and thus, there may have been other health consequences of poverty interfering with fecundability. In rural Bangladesh, young wives would have had gone through menarche to start living with their husbands (Field & Ambrus, 2008); therefore, it is unlikely that married girls in our study population remained premenarchal. This inference is supported by a study showing that 99% of 12‐ to 19‐year‐old married girls in the same research site were postmenarcheal (Rah et al., 2009). The younger aged girls in this study were therefore selected as early or average maturers, which may be a factor in their getting married.

In summary, in rural Bangladesh, where strong social expectation for childbearing soon after marriage is still prevalent, thin adolescent newlyweds were less likely to become pregnant, and among those who did, took longer to become pregnant than better nourished young women. Although the generalizability of our findings to other rural or urban contexts remains to be investigated, plausible biological mechanisms exist by which chronic undernutrition may suppress reproductive competence, whereas biosocial influences cannot be ruled out. As an important public health measure, in Bangladesh, the government has established a constitutional act to raise girls' age of marriage to 18 years or older (Government of the People's Republic of Bangladesh Ministry of Women and Child Affairs, 2017), although the law contains a potential loophole by allowing young girls to enter marriage under special circumstances, such as parental consent. As cultural change must accompany such a law, child marriage remains common in Bangladesh (National Institute of Population Research and Training & ICF, 2019). Thus, although efforts to improve nutritional status of girls remain a public health mandate in any undernourished population, effective policies and education are likely required to reduce adolescent marriage and motherhood. Doing so may improve maternal growth, nutritional status (Rah et al., 2008), and birth outcomes (Kozuki et al., 2013) and help interrupt a persistent intergenerational cycle of undernutrition, and thus improve health and quality of life of young women, in rural South Asia.

## CONFLICTS OF INTEREST

None of the authors have conflicts of interest to disclose.

## CONTRIBUTIONS

JH analysed the data, interpreted the results, and drafted the manuscript; KPW, KJS, and PC provided critical revision to the manuscript for important intellectual content; KPW, MR, ABL, RDWK, and PC conceived and designed the study; AAS, MR, ABL, HA, and BU acquired the data; LS‐FW provided statistical support; AAS, MR, ABL, HA, and BU provided administrative support; KPW, AAS, MR, ABL, HA, BU, KJS, and PC supervised the study.

## Supporting information


**Table S1** Adjusted hazard ratio (HR) of pregnancy in Bangladeshi adolescent newlyweds by early postnuptial mid‐upper arm circumference (MUAC) being ≤ 20.0 vs > 20.0 cm during 5‐6 years of observation of the JiVitA‐1 trial, 2001‐2002 enlisted cohort
**Table S2** Adjusted hazard ratio (HR) of pregnancy in Bangladeshi adolescent newlyweds by early postnuptial mid‐upper arm circumference (MUAC) being ≤ 19.0 vs > 19.0 cm during 5‐6 years of observation of the JiVitA‐1 trial, 2001‐2002 enlisted cohort
**Table S3** Distribution of entry into the pregnancy surveillance system of the JiVitA‐1 trial by calendar month and early postnuptial mid‐upper arm circumference (MUAC) of Bangladeshi adolescent newlyweds, 2001‐2002 enlisted cohort^a^
**Table S4** Time to censorship (mean ± SD) by early postnuptial mid‐upper arm circumference (MUAC) of Bangladeshi adolescent newlyweds who never became pregnant during 5‐6 years of observation of the JiVitA‐1 trial, 2001‐2002 enlisted cohort
