# Supplementary material for: Thinness and fecundability: Time to pregnancy after adolescent marriage in rural Bangladesh
Source: Matern Child Nutr. 2020 Mar 24;16(3):e12985. doi: 10.1111/mcn.12985 (PMC7296800; doi:10.1111/mcn.12985)
Supplement: Supplementary file 1 — Table S1 Adjusted hazard ratio (HR) of pregnancy in Bangladeshi adolescent newlyweds by early postnuptial mid‐upper arm circumference (MUAC) being ≤ 20.0 vs > 20.0 cm during 5‐6 years of observation of the JiVitA‐1 trial, 2001‐2002 enlisted cohort Table S2 Adjusted hazard ratio (HR) of pregnancy in Bangladeshi adolescent newlyweds by early postnuptial mid‐upper arm circumference (MUAC) being ≤ 19.0 vs > 19.0 cm during 5‐6 years of observation of the JiVitA‐1 trial, 2001‐2002 enlisted cohort Table S3 Distribution of entry into the pregnancy surveillance system of the JiVitA‐1 trial by calendar month and early postnuptial mid‐upper arm circumference (MUAC) of Bangladeshi adolescent newlyweds, 2001‐2002 enlisted cohorta Table S4 Time to censorship (mean ± SD) by early postnuptial mid‐upper arm circumference (MUAC) of Bangladeshi adolescent newlyweds who never became pregnant during 5‐6 years of observation of the JiVitA‐1 trial, 2001‐2002 enlisted cohort [file MCN-16-e12985-s001.docx]

| **Supplementary Table 1** Adjusted hazard ratio (HR) of pregnancy in Bangladeshi adolescent newlyweds by early postnuptial mid-upper arm circumference (MUAC) being ≤ 20.0 vs > 20.0 cm during 5-6 years of observation of the JiVitA-1 trial, 2001-2002 enlisted cohort | | | | |
| --- | --- | --- | --- | --- |
|  | Pregnancies (*n*) | HR | 95% CI | *p* |
| MUAC at enlistment (cm) |  |  |  | < 0.001 |
| > 20.0 (*n* 5,040)^a^ | 4,053 | 1.00 |  |  |
| ≤ 20.0 (*n* 474)^a^ | 343 | 0.79 | 0.70, 0.88 |  |
| Family planning practice at enlistment |  |  |  | 0.630 |
| No | 3,929 | 1.00 |  |  |
| Yes | 467 | 0.98 | 0.89, 1.08 |  |
| Age at marriage (years) | 4,396 | 1.06 | 1.04, 1.08 | < 0.001 |
| Year of marriage |  |  |  | 0.020 |
| 2001 | 1,354 | 1.00 |  |  |
| 2002 | 3,042 | 0.92 | 0.86, 0.99 |  |
| Season of marriage |  |  |  | 0.076 |
| Monsoon | 1,919 | 1.00 |  |  |
| Post-monsoon | 829 | 0.99 | 0.91, 1.07 |  |
| Post-harvest | 914 | 1.08 | 1.00, 1.17 |  |
| Hot and humid summer | 734 | 0.95 | 0.87, 1.04 |  |
| ^a^Missing values > 20.0 cm: *n*=1; ≤ 20.0 cm: *n*=1, both due to missing data in family planning practice covariate | | | | |

| **Supplementary Table 2** Adjusted hazard ratio (HR) of pregnancy in Bangladeshi adolescent newlyweds by early postnuptial mid-upper arm circumference (MUAC) being ≤ 19.0 vs > 19.0 cm during 5-6 years of observation of the JiVitA-1 trial, 2001-2002 enlisted cohort | | | | |
| --- | --- | --- | --- | --- |
|  | Pregnancies (*n*) | HR | 95% CI | *p* |
| MUAC at enlistment (cm) |  |  |  | 0.012 |
| > 19.0 (*n* 5,363)^a^ | 4,287 | 1.00 |  |  |
| ≤ 19.0 (*n* 151)^a^ | 109 | 0.78 | 0.65, 0.95 |  |
| Family planning practice at enlistment |  |  |  | 0.734 |
| No | 3,929 | 1.00 |  |  |
| Yes | 467 | 0.98 | 0.89, 1.08 |  |
| Age at marriage (years) | 4,396 | 1.06 | 1.04, 1.08 | < 0.001 |
| Year of marriage |  |  |  | 0.026 |
| 2001 | 1,354 | 1.00 |  |  |
| 2002 | 3,042 | 0.92 | 0.86, 0.99 |  |
| Season of marriage |  |  |  | 0.081 |
| Monsoon | 1,919 | 1.00 |  |  |
| Post-monsoon | 829 | 0.98 | 0.90, 1.07 |  |
| Post-harvest | 914 | 1.07 | 0.99, 1.16 |  |
| Hot and humid summer | 734 | 0.95 | 0.87, 1.04 |  |
| ^a^Missing values > 19.0 cm: *n*=1; ≤ 19.0 cm: *n*=1, both due to missing data in family planning practice covariate | | | | |

| **Supplementary Table 3** Distribution of entry into the pregnancy surveillance system of the JiVitA-1 trial by calendar month and early postnuptial mid-upper arm circumference (MUAC) of Bangladeshi adolescent newlyweds, 2001-2002 enlisted cohort^a^ | | | |
| --- | --- | --- | --- |
|  | MUAC > 21.5 cm (*n* 3,746) | MUAC ≤ 21.5 cm (*n* 1,770) | Total (*n* 5,516) |
| April 2001 | 2 (0.1) | 1 (0.1) | 3 (0.1) |
| May 2001 | 7 (0.2) | 1 (0.1) | 8 (0.2) |
| June 2001 | 40 (1.1) | 28 (1.6) | 68 (1.2) |
| July 2001 | 150 (4.0) | 69 (3.9) | 219 (4.0) |
| Aug 2001 | 260 (6.9) | 125 (7.1) | 385 (7.0) |
| Sep 2001 | 234 (6.3) | 107 (6.1) | 341 (6.2) |
| Oct 2001 | 142 (3.8) | 82 (4.6) | 224 (4.1) |
| Nov 2001 | 124 (3.3) | 55 (3.1) | 179 (3.3) |
| Dec 2001 | 137 (3.7) | 73 (4.1) | 210 (3.8) |
| Jan 2002 | 244 (6.5) | 127 (7.2) | 371 (6.7) |
| Feb 2002 | 274 (7.3) | 119 (6.7) | 393 (7.1) |
| Mar 2002 | 246 (6.6) | 114 (6.4) | 360 (6.5) |
| Apr 2002 | 205 (5.5) | 98 (5.5) | 303 (5.5) |
| May 2002 | 167 (4.5) | 71 (4.0) | 238 (4.3) |
| Jun 2002 | 379 (10.1) | 186 (10.5) | 565 (10.2) |
| Jul 2002 | 446 (11.9) | 182 (10.3) | 628 (11.4) |
| Aug 2002 | 311 (8.3) | 154 (8.7) | 465 (8.4) |
| Sep 2002 | 174 (4.6) | 80 (4.5) | 254 (4.6) |
| Oct 2002 | 122 (3.3) | 67 (3.8) | 189 (3.4) |
| Nov 2002 | 37 (1.0) | 16 (0.9) | 53 (1.0) |
| Dec 2002 | 45 (1.2) | 15 (0.9) | 60 (1.1) |
| *p* = 0.793  ^a^Percentages may not equal to 100% due to rounding | | | |

| **Supplementary Table 4** Time to censorship (mean ± SD) by early postnuptial mid-upper arm circumference (MUAC) of Bangladeshi adolescent newlyweds who never became pregnant during 5-6 years of observation of the JiVitA-1 trial, 2001-2002 enlisted cohort | | | | | | | |
| --- | --- | --- | --- | --- | --- | --- | --- |
|  | MUAC > 21.5 cm | | MUAC ≤ 21.5 cm | | Total | | *p* |
|  | *n* | mean ± SD | *n* | mean ± SD | *n* | mean ± SD |  |
| Time to censorship (weeks) | 690 | 165.2 ± 93.7 | 429 | 166.7 ± 96.4 | 1,119 | 165.8 ± 94.7 | 0.799 |
|  | | | | | | |  |
